# Establishment and Fractionation of Metastatic Axillary Lymph Node Cell Suspension for Determination of Protein Expression Levels of Nuclear cFOS and Cytosolic TGFβ1 from Breast Cancer Patients

**DOI:** 10.1186/s12575-022-00167-x

**Published:** 2022-06-04

**Authors:** Vesna Ivanović, Nasta Dedović-Tanić, Zorka Milovanović, Bratislav Stojiljković, Miroslav Demajo, Vesna Mandušić

**Affiliations:** 1 Department for Radiobiology and Molecular Genetics, Institute of Nuclear Sciences “Vinča”, University of Belgrade, Belgrade, 11001 Serbia; 2 State University of Novi Pazar, Department of Natural Sciences and Mathemathics, Novi Pazar, Serbia; 3 Institute of Oncology and Radiology of Serbia, Belgrade, Serbia; 4 Oncology Institute of Vojvodina, Sremska Kamenica, Serbia

**Keywords:** Axillary lymph nodes, cFOS, TGFβ1, FNCS, Fractionated Nodal Cell Suspension

## Abstract

**Background:**

Metastatic Axillary Lymph Node (mALN) status is currently the most important prognostic factor in the management of primary breast cancer (BC). Thus, development of specimens which enable identification of new mALN markers, involved in the progression of the disease, are of considerable interest. The specific aim of this work was to describe the method of establishment of Metastatic Axillary Nodal Cell Suspension and its fractionation, termed Fractionated Nodal Cell Suspension (FNCS), into nuclear and cytosolic extracts to enable determination of protein expression levels of nuclear cFOS and cytosolic Transforming Growth Factor β1 (TGFβ1) in BC patients.

**Results:**

To standardize the procedure, HeLa cells were successfully fractionated into nuclear/cytosolic extracts with confirmed presence of nuclear cFOS and cytosolic TGFβ1 proteins. Subsequently, the ALN Cell Suspension specimens were obtained and further fractionated from a pilot sample of six ALN tissue pairs, mALN versus autologous normal ALN (nALN), dissected from invasive BC patients. The mALN/nALN results revealed overexpression of both nuclear cFOS and cytosolic TGFβ1 protein levels. However, only the TGFβ1 data exhibited statistically significant overexpression, which was proportional to the respective values of mALN diameter of tumor deposits.

**Conclusions:**

Detailed protocol for establishment and fractionation of mALN cell suspension specimens, termed FNCS, into nuclear and cytosolic extracts is here described for the first time. This approach might be a convenient ex vivo model for simultaneous analysis of protein, RNA and DNA biomarkers from nuclear/cytosolic extracts of the same mALN tissue sample. It might have potential to enable, in the age of genomics and personalized medicine, an identification of novel mALN biomarkers and thus improve the screening, diagnosis and prognosis of invasive BC.

## Background

Breast cancer (BC)^Abb^ is a leading malignancy in women worldwide [1] with extremely heterogeneous cell morphology [2, 3] including more than 20 distinct subtypes that differ genetically and clinically [4]. Distant metastasis is the main cause of death in BC patients. Axillary lymph nodes (ALN) are the main doorway for tumor cell escape from the primary site to other regions of the body [5]. Consequently, metastatic ALN (mALN) are considered the most important prognostic factors and powerful source of biomolecules that may become reliable metastatic biomarkers. In spite of that, very few studies have been conducted to identify BC biomarkers associated with the ALN metastasis of BC. Therefore, inclusion of new mALN molecular biomarker profiles has been proposed to predict nodal status at the time of BC diagnosis [6].

Considerable research attention has been focussed on a role of deregulation of Transforming Growth Factor β1 (TGFβ1) as tumor promoter step favoring BC invasion and metastasis [7]. Moreover, accumulating evidence shows that FOS transcription factor binding motifs are critical for the regulation of TGFβ1 expression [8]. Thus, cFOS elevation may have utility as a complementary candidate biomarker of BC invasiveness, co-expressed with TGFβ1. Consequently, we have previously proposed that cFOS and TGFβ1 proteins may be considered as a pair of biomarkers of an early assessment of invasive BC [7, 8], providing adequate invasive BC specimens are available. In the past, primary tumor tissue had been fractionated into nuclear [9] or cytosolic [10, 11] extract to assess specific biomarkers of interest. Until recently, however, the protocol for fractionation of mALN nuclear and cytosolic extracts has not been available, presumably due to the specific tough, fibrous nature of mALN tissue.

To date, diameter of tumor deposits and proliferation index Ki67 [6, 12, 13] are the most prominent clinically used features of mALN. Both parameters are detected by routine histology [14] involving the tissue paraffin blocks of 4-μm slices for each node [15, 16] and their staining with either Haematoxylin and Eosin (H&E) or Immunohistochemistry (IHC), respectively [17]. Although indispensable, the histology methodology imposes some limitations [16]. Namely, in a significant portion of cases, due to clustered spacial distribution of tumor cells within a lymph node, the histology sectioning generates sampling errors leading to false negative mALN sections [18]. Consequently, the pool of biomarkers in various slices may be different. To overcome this problem, Edwards and co-workers [19] introduced mALN Cell Suspension as new specimens thereby covering the whole content of entire mALN. Based on this mALN specimen source, we developed the method, termed Fractionation of Nodal Cell Suspension (FNCS), which includes the mALN Cell Suspension approach and its fractionation into nuclear and cytosolic extracts to be suitable for determination of protein expression levels of respective native proteins. Using this experimental design, we have previously observed overexpression of TGFβ1 protein in cytosolic extracts of mALN [7]. Likewise, we encountered a case of an advanced Triple Negative Breast Cancer (TNBC) patient with overexpressed both cytosolic TGFβ1 and nuclear cFOS proteins as a pair of mALN biomarkers for an early assessment of TNBC poor prognosis [8]. However, in above mentioned studies [7, 8], apart from the outlined methods used, specific experimental protocols were not described. Having in mind that the FNCS design might help to generate an important predictive tool suitable for comparative analysis of individual patients in present era of genomics and personalized medicine [2], we undertook the present study. The main goal was to describe the full methodology of establishment and fractionation of mALN Cell Suspension thus providing FNCS specimens of nuclear and cytosolic extracts and determination of protein expression levels of respective cFOS and TGFβ1.

**Fig. 1:**
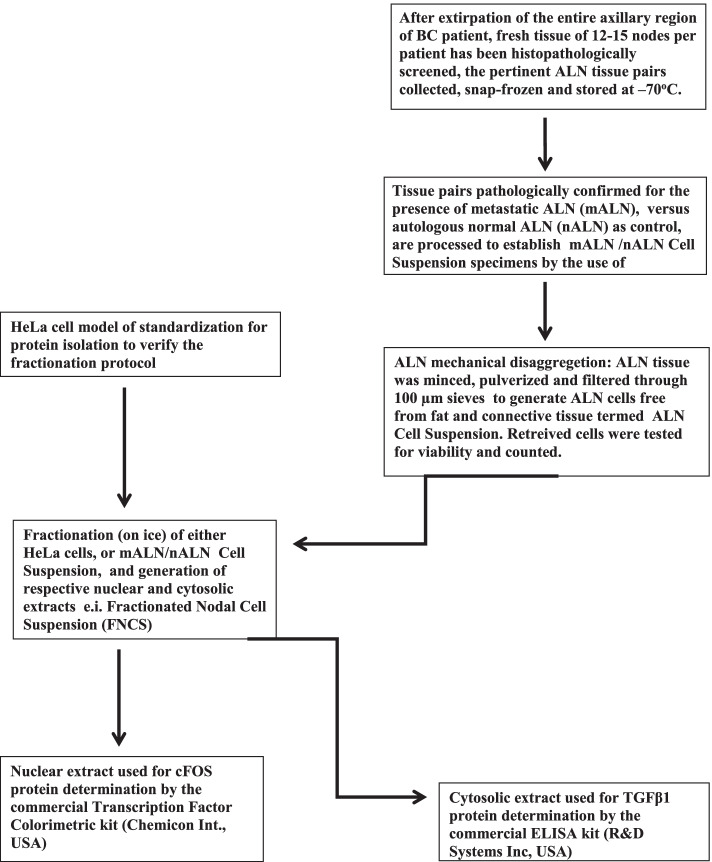
Workflow of the experimental design for the present study, to establish and fractionate Metastatic Nodal Cell Suspension specimen termed FNCS, which enables determination of protein expression level of nuclear cFOS and cytosolic TGFβ1

The workflow of the this study is presented in Fig. 1. After extirpation of the entire axillary lymph node of BC patient and selection of the pertinent nodes (mALN/nALN pairs), the following steps, included in the experimental design, were: *i*) mechanical disaggregation of ALN, chopped and filtered through 100 μm sieve devices, to obtain mALN cell suspension free from fat and connective tissue (mALN Cell Suspension); *ii)* model protocol of HeLa cell fractionation into nuclear and cytosolic extracts, confirmed by respective internal controls, to be implemented on mALN Cell Suspension to obtain FNCS specimens and generate nuclear/cytosolic extracts; *iii)* determination of protein expression level of nuclear cFOS and cytosolic TGFβ1 by ELISA; *iv)* correlation of the respective cFOS and TGFβ1 biomarker levels with mALN diameter of tumor deposits for each BC patient.

Since mALN tissue sample is heterogeneous in terms of its cell content (including: various BC malignant clones, fibroblasts, macrophages, lymphocytes etc.) this protocol enables the use of autologous normal ALN (nALN) of each patient as an optimal choice of negative control. Taken together, this study provides tools to researchers termed FNCS, in which mALN and nALN tissue samples are used as initial ex vivo materials, to follow the protocol “from tough mALN/nALN tissue, through mALN/nALN Cell Suspension, to fractionationate the nuclear/cytosolic extracts” and enable ELISA determination of respective protein biomolecules. The method provides considerable advantages, when compared to current pathohistological BC diagnostics which is, during routine examination, rather limited to defined slices which cannot cover the complete volume of the nodal tissue.

## Results

### Hela Cell In Vitro Model for Fractionation of Nuclear and Cytosolic Extracts and Determination of Respective Protein Expression Levels of cFOS and TGFβ1

**Fig. 2:**
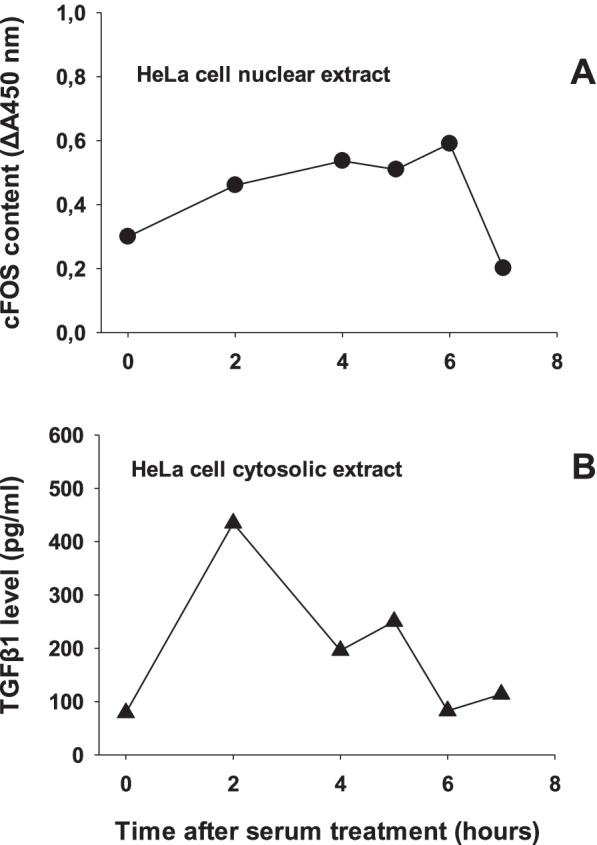
HeLa cell fractionation model illustrated by time curves after serum treatment for: **A** nuclear cFOS protein, presented by black circles (**●**), and **B** cytosolic TGF β1 protein, presented by black triangles (**▲**). The extracts were obtained by fractionation of growing HeLa cells as described in Methods. All the time points were simultaneously processed and assayed in a single cFOS or TGF-β1 ELISA microplate. Each point was run in duplicate and represents the mean value of two wells. The intra-assay (*n* = 6) and inter-assay (*n* = 4) reproducibility was 7 and 15%, respectively

**Fig. 3:**
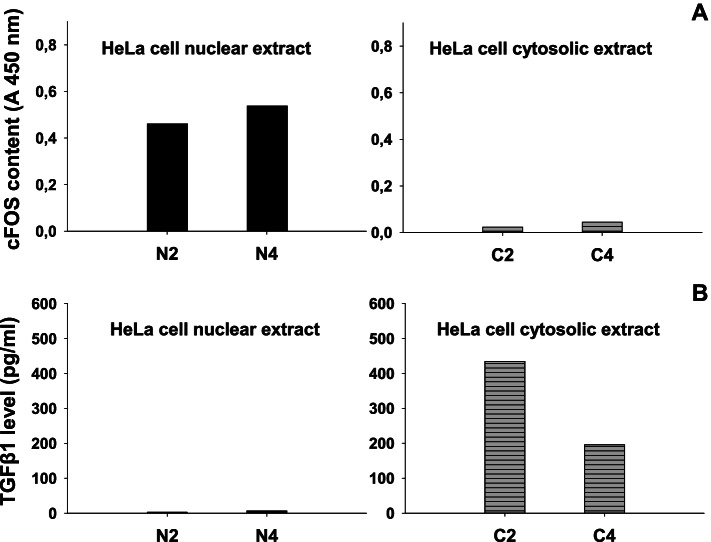
Test of internal control: to verify that the detected cFOS and TGFβ1 markers were derived from HeLa cell nucleus and cytoplasm, respectively. Histograms represent the two time points (t = 2 & t = 4 hrs) selected from time curves presented in Fig. 2 and the two markers measured in nuclear extracts (designated as N2& N4) and cytosolic extracts (designated as C2&C4): **A** cFOS oncoprotein levels in nuclear versus cytosolic HeLa cell extracts and **B** TGF-β1 levels in nuclear versus cytosolic HeLa cell extracts

In order to save the precious ALN tissue, the experimental approach included the use of HeLa cell culture to confirm the fractionation protocol described in Methods. Historically, HeLa cell fractionation into nuclear and cytosolic fractions had been introduced in molecular biology research about four decades ago [20]. Moreover, previous studies have established that transcription factors including cFOS are nuclear markers of mammalian cells as demonstated in HeLa cell nuclear extracts [21]. Likewise, the HeLa cell cytoplasmic extracts have been used to study cytoplasmic proteins [22]. Based on these facts we have monitored the time courses of serum-induced nuclear cFOS and cytosolic TGF β1 proteins. The results, presented in Fig. 2 reveal that nuclear cFOS reached and kept the plateau values between 4 and 6 hours after serum treatment (Fig. 2A). Accordingly, as recommended by the manufacturer [21], the 4 hr. nuclear extract point was used as cFOS positive control sample in subsequent ALN measurements of cFOS. To verify the usefulness of the selected fractionation protocol on cytosolic TGF-β1 detectability, autologous time points of HeLa cytosolic extracts were analyzed by TGF-β1 ELISA. The results in Fig. 2B illustrate that the cytosolic TGF-β1 was induced rapidly and reached maximal value at 2 hours after serum treatment, followed by continuous decrease in values. To verify whether the detected cFOS and TGF-β1 were derived from HeLa cell nucleus and cytoplasm, respectively, we performed the following experiment. The nuclear (N) and cytosolic (C) fraction of the two time points (t = 2 and t = 4 hrs) in Fig. 2 have been analyzed for the presence of either cFOS in cytosolic extracts, or TGF-β1 in nuclear extracts. The data presented in Fig. 3 reveal that neither was cFOS detected in cytosolic extracts, nor was TGF-β1 detected in nuclear extracts of HeLa cells (Fig. 3). Taken together, these results indicate that the selected fractionation protocol has successfully separated cytosolic from nuclear fraction of HeLa cells and preserved the native nature of the analyzed proteins.

### Description of ALN Tissue Morphology

**Fig. 4:**
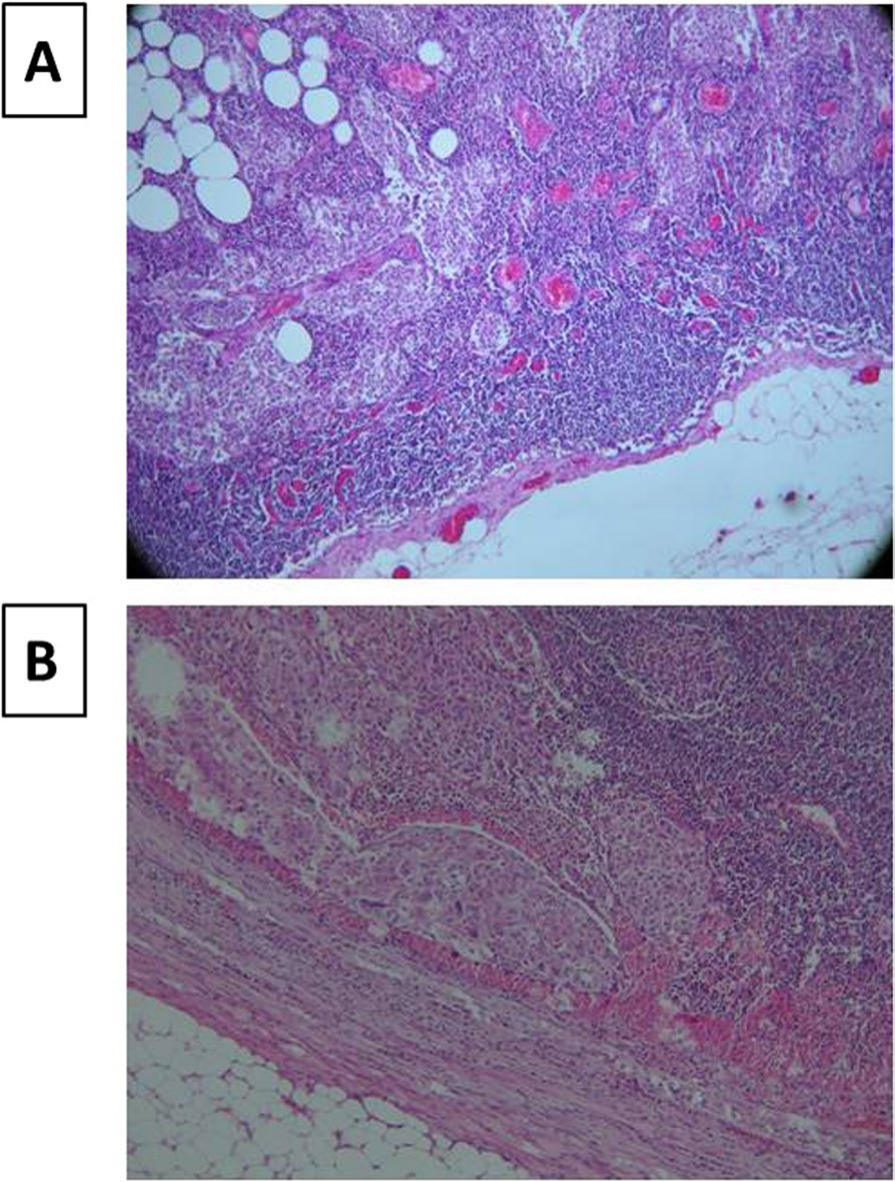
Comparative images of H&E stained morphology for axillary lymph nodes of patient #1 in Table 1: **A** Normal ALN tissue (nALN) as control, without metastatic deposits and **B** Autologous metastatic ALN (mALN) with near total replacement of lymph nodal tissue by nodules of metastasis (black dots)

These findings encouraged us to morphologically characterize mALN tissue of pilot samples of six patients with clinical characteristics of invasive BC. These patients, labeled 1-6, are described in Table 1. In addition, respective normal ALN (nALN) tissue was selected from each patient as an optimal choice of negative control tissue. Subsequent H&E staining was performed on pairs of surgical tissue for each patient. Figure 4 illustrates cell morphology of ALN tissue pairs for patient #1 in Table 1. The results for control nALN, reveal stasis, multifocal lipomatosis and sinus histiocytosis of the lymph node without metastatic deposits (Fig. 4A). On the other hand, in case of mALN (Fig. 4B) the morphology reveals large malignant polygonal cells with eosinophilic cytoplasm and hyperchromatic nuclei. These cytology features correspond to primary breast cancer.

**Table 1:** Clinical and pathohistological characteristics of BC patients from whom metastatic ALNs were dissected

Characteristics	Patients’ number					
	1	2	3	4	5	6
**Age at surgery**	74	39	70	52	48	66
**Menopausal status**	Post	Pre	Post	Post	Pre	Post
**ER**^a^	0	0	8	8	7	8
**PR**	0	0	7	0	0	8
**HER2**	0	0	1^a^	0	1+	1+
**TNBC status**	TNBC	TNBC	nonTNBC	nonTNBC	nonTNBC	nonTNBC
**Histological Grade**^b^	III	II	II	II	II	II
**Type AJCC**^c^	ILC/ IAC	IDC	ILC	ILC	ILC	ILC
**Clinical Stage**	IIIB	IIIA	IIIA	IIIA	II	II
**Lymph node status**	3^d^/8	4/18, pni+,^f^	7/13, pni+,^f^	7/14	1/8, pni+,^f^	1/15
**pTNM**	pT4d^e^, N2, M0	pT1a, N2a, M0	pT2, N2a, M0	pT: pTU1^g^: 2, N2a, M0 pTU2^g^: 1c	pT1c, N1a, M0	pT1c, N1a, M0

^a^Grading scale used to evaluate ER and PR ranged from 0 to 8. The cut-off value was 3, above which the markers were considered positive as proposed previously^20^

^b^Assessment of histological grade of primary breast tumor was based on Scarff-Bloom-Richardson system (I-III)

^c^Patients were staged according to the American Joint Committee on Cancer (AJCC) 6th edition staging manual [23]

^d^Metastasis observed in 3 out of 8 ALNs

^e^large primary tumor, “d”, with malignant infiltration of surrounding skin

^f^pni+, malignant infiltration of perinodal adipose tissue

^g^TU1 and TU2 signify two primary tumor lesions diagnozed simultaneously on the same breast of the indicated BC patient

### Individualized Comparative Analysis of Diameter of Tumor Deposits Versus Respective ALN Nuclear cFOS and Cytosolic TGF-β1 Levels

**Fig. 5:**
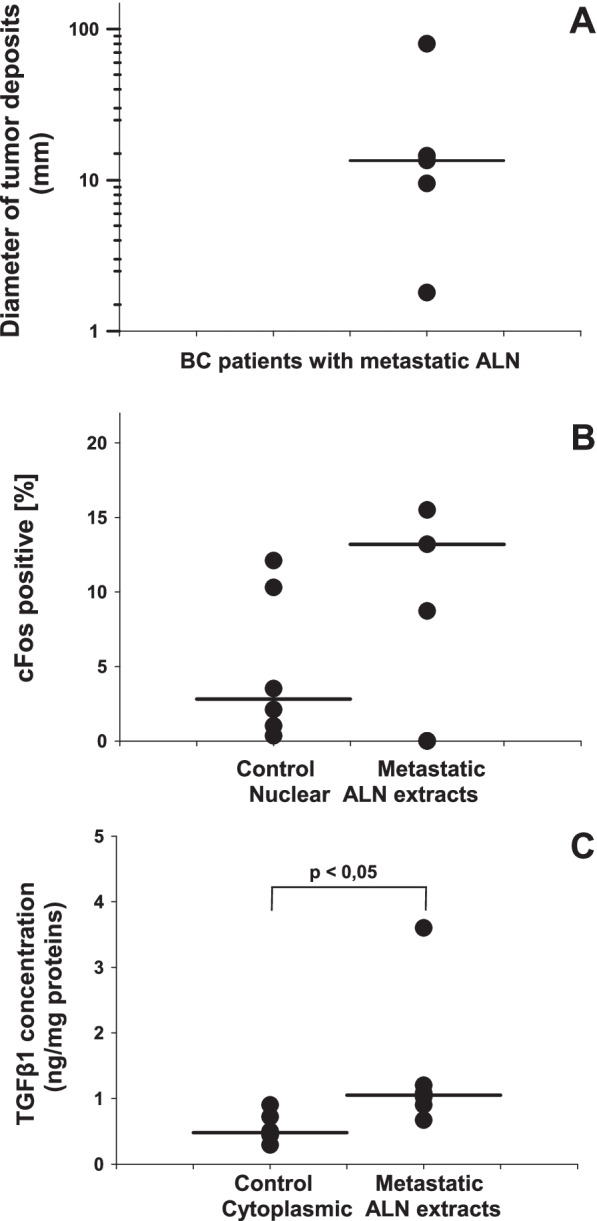
Comparative scatter diagrams of ALN biomarkers of BC patients described in Tables 1 & 2. **A** diameter of tumor deposits of metastatic ALN; horizontal bar represent median value; Median 13.5; Mean 23.86 ± S.E.M. 12.74; number of samples = 5. **B** Levels of nuclear cFOS detected in FNCS-derived extracts of nonmalignant ALN tissue as a control versus that of the autologous metastatic ALN tissue; horizontal bars represent median values for nALN versus mALN: Control: Median 2.81; Mean 4.89 ± SEM 1.88; number of samples = 6; Metastatic: Median 13.2; Mean 12.47 ± SEM 1.63; number of samples = 3; Median significance *p* > 0.05. Here no significant difference between C and M. **C**. Levels of cytosolic TGFβ1 in FNCS-derived extracts of nomal ALN tissue as a control versus that of the autologous metastatic ALN tissue: horizontal bars represent median values; p, level of significance for nALN versus mALN: Control: Median 0.48; Mean 0.55 ± SEM 0.08; number of samples = 6; Metastatic: Median 1.05; Mean 1.41 ± SEM 0.40; number of samples = 6. Median significantly different, *p* < 0.05

The individualized diameter of tumor deposits (range: 80-1.8 mm, median value: 13.5 mm) assessed from the H&E staining of formalin-fixed, paraffin-embedded slices, are presented in Fig. 5A and Table 2.

**Table 2:** Individual parameter values measured in the selected single metastatic ALN (mALN) for each of the BC patient presented in Table 1 and described in Methods and Fig. 4

Parameter	Patients’ number					
	1	2	3	4	5	6
**Diameter of tumor deposits**^a^ **(mm)**	80.0^b^	9.5	14.5	13.5	1.8	n.a.^d^
**% of malignant cells**	80%	100%	100%	100%	30%	n.a.
**Presence of micrometastasis**^c^	none	none	none	none	positive	n.a.
**Initial tissue weight** **(mg)**	790	720	190	660	630	530
**Total viable cell number (× 10**^**− 6**^**)**	18.6	3.6	5.2	9.0	3.8	3.9
**Nuclear protein yield (mg)**	3.2	0.6	1.0	1.7	1.1	0.8
**Cytosolic protein yield (mg)**	6.5	1.2	1.7	2.5	0.9	1.2
**cFOS level (%)**	15.50	8.75	13.20	0.00	0.00	0.00
**TGFβ1 level (ng/mg protein)**	3.60	1.20	0.90	1.02	0.67	1.08

^a^The size of tumor deposits was assessed from the H&E slices of formalin-fixed, paraffin-embedded ALN tissue

^b^“mALN1” essentially exhibited the conglomerate size: 80 × 50 × 40 mm, with malignant infiltration of surrounding ALN tissue

^c^observed morphologically

^d^not available due to much too small ALN size and the entire “mALN 6” was used for cFOS and TGFβ1 analysis

However, H&E staining does not always provide enough contrast to differentiate all tissues and cellular structures [24], therefore in these cases more specific stains by IHC method are used. Likewise, quantifying the IHC stains is also limited, subject to human error, and not standardized worldwide [25]. Due to these disadvantages, the protocol of FNCS method was implemented on pairs of ALN tissues from patients 1-6 in Table 1 as described in Methods. The quantitative parameters obtained from these experimental procedures are summarized in Table 2.

For each patient labeled consecutively 1-6, they include: the initial tissue weight (range: 790-190 mg), total viable cell number (range: 18.6-3.6 × 10^6^), yields of total nuclear protein (range: 3.2-0.6 mg) and total cytosolic protein (range: 6.5-0.9 mg) of the metastatic ALN. Respective measurements of normal ALN values of the same parameters revealed similar ranges and yields as described in Table 2. (data not shown).

The levels of cFOS and TGFβ1 expression in the mALN/nALN tissue pairs of respective FNCS specimens are presented in Fig. 5B & C.and Table 2. The data in Fig. 5B indicate that cFOS levels in mALN nuclear extracts (median value:13.20, *n* = 3) were elevated when compared with those of nALN (median value: 2.81%, *n* = 6), although statistical significance (*p* > 0.05) was not observed. On the other hand, the data in Fig. 5C show that TGF β1 levels in mALN cytoplasmic extracts (median value: 1.05 ng/mg protein, *n* = 6) were significantly (*p* < 0.05) elevated when compared with those of nALN cytoplasmic extract (median value: 0.48 ng/mg protein, *n* = 6) which was proportional to the size of respective mALN tumor deposits (Table 2). As a consequence, they suggest that TGF β1 overexpression is associated with the presence of metastatic cells in the ALN-positive tissue specimens. Moreover, Fig. 5A and C illustrate complementary role of routine histology and FNCS analysis in confirmation of TGF β1 as an individual putative metastatic biomarker, suggesting that both methods are beneficial in diagnostics of invasive BC patients.

## Discussion

It is now well established that accurate staging of the ALN for metastatic disease is critical in deciding the appropriate management of BC patients. Metastasis to the axillary nodes is the earliest sign of the BC metastatic spread and this process occurs at a higher rate than any single distant organ metastasis [26]. However, probability of false negatives due to the routine ALN histology sectioning underestimates ALN positivity in a significant proportion of cases (for details see Introduction). Likewise, pathologists face difficulties in detecting ALN micrometastasis. In these cases they use IHC staining and multiple sectioning of each node which is time consuming and expensive [19]. With these justifications, in the present study we described in details the establishment and fractionation protocol of mALN cell suspension into nuclear and cytosolic extract, termed Fractionated Nodal Cell Suspension (FNCS) as bench flow procedure to investigate invasive BC biomarkers and possibly assess the ALN status as the subsidiary tool in routine clinical use.

Currently, available model systems for pre-clinical metastatic BC research, include primary cell culture, immortalized cell lines, mouse xenographts [27] and organoid technology [28, 29]. Although essential for discovery, development, and testing of new therapies, these models have both advantages and disadvantages [17]. In spite of that, very few studies have been conducted to identify BC biomarkers associated with the ALN metastasis of BC. Among these, mammaglobin has been identified for the detection of metastatic ALN in BC [30], as detected by absolute quantitative real-time reverse transcription-PCR (qRT-PCR). Therefore, inclusion of additional new mALN molecular biomarker profiles is needed to predict nodal status at the time of BC diagnosis [6].

Here we describe the design of a potentially new BC specimen, termed Fractionated Nodal Cell Suspension to establish and fractionate mALN Cell Suspension based on disaggregation of dissected mALN tumor tissue, preparation of mALN Cell Suspension, its further fractionation and determination of protein expression level of nuclear cFOS and cytosolic TGFβ1 from the same ALN sample. Although cFOS is elevated but not statistically significant, our TGFβ1 results reveal statistically significant overexpression in respective cytosolic extracts, which was proportional to the size of respective mALN tumor deposits. Major limitation of this data pertains to the small sample size. Nevertheless, our findings imply that the TGFβ1 overexpression is associated with the presence of metastatic BC cells. Large scale studies, however, are necessary to confirm the conclusion.

Taken together, the FNCS method may facilitate simultaneous comparative analysis of other protein, RNA and DNA biomarkers from the same ALN tissue sample. This might provide very powerful measurements which allow direct genotypic and phenotyping correlations [26]. Along this line, the main characteristics of cells in FNCS, such as the expression of ER, PR and HER2, may be analyzed to presumably generate surrogate ALN samples to explore whether they match the histopathologic characteristics of the corresponding BC patients. Alternatively, we expect advanced application of the FNCS model system which might include an enrichment of a particular malignant clone content starting with mALN Cell Suspension specimen and clonal evolution during the migration e.i. metastasis. Namely, mALN Cell Suspension specimens being heterogeneous in the cell population (for details see Introduction) could be further separated by selective gradient to obtain individual cell types [17] to be further sorted out [31] by Fluorescence Activated Cell Sorter (FACS). After these steps, samples of homogeneous tumor cell clones are to be obtained. The proposed FNCS design might be advantageous, when compared to highly sophisticated and expensive Laser Capture Microdissection (LCM) which is based on the small number of cells consequently yielding low RNA and DNA amounts. Furthermore, described FNCS design provides an excellent sample source for investigating molecular changes during the disease progression.

## Conclusion

Our study provides, for the first time, detailed description of the experimental method for establishment and fractionation of Metastatic Axillary Nodal Cell Suspension into nuclear and cytosolic extracts, termed FNCS, for determination of protein expression levels of respective cFOS and TGFβ1. This convenient procedure might be a valuable tool in pre-clinical research of other invasive BC biomarkers. It has considerable advantages, when compared to pathohistological ALN diagnostics, since one need not worry about false negatives and one can reliably quantify treatment-dependent comparative biomarker levels of individual patients. Thus, in the present era of genomics and personalized medicine, the described FNCS method might facilitate the identification of new mALN biomarkers and improve the screening, diagnosis and prognosis of invasive BC.

## Methods

### Patients and ALN Assessment

Twelve consecutive female BC patients, attending the hospitals within 6 weeks (from August 24, to October 04, 2007), underwent extirpation of the entire axillary region. Out of this fresh tissue, 12-15 nodes per patient have been screened histopathologically and the study cohort of 6 ALN-positive patients was obtained and presented in Table 1. None of these patients were previously treated. The proposed prospective study had received the Institutional Review Board approval and a written informed consent was obtained from each woman according to the National Health Regulation. For each BC patient, a fresh tissue of single metastatic ALN with maximal size of tumor deposits was divided in half. The first half underwent routine histopathological examination using cryotome-cut frozen sections stained by H&E. After confirmation of the presence of metastatic deposits, the second half of fresh tissue, selected for this research, was immediately snap-frozen and stored at − 70 °C, within the shelf time of 2 months. Simultaneously with mALN, autologous, histopathologically verified, normal ALN control (nALN) was collected. These pairs of tissue samples, after defrosting, were subjected to disaggregation protocol (see below) to establish respective ALN Cell Suspensions and further fractionation to obtain FNCS specimens for subsequent cFOS and TGFβ1 determination.

### Protocol for Disaggregation of mALN Tissue and Establishment of mALN Cell Suspension Specimen

**Fig. 6:**
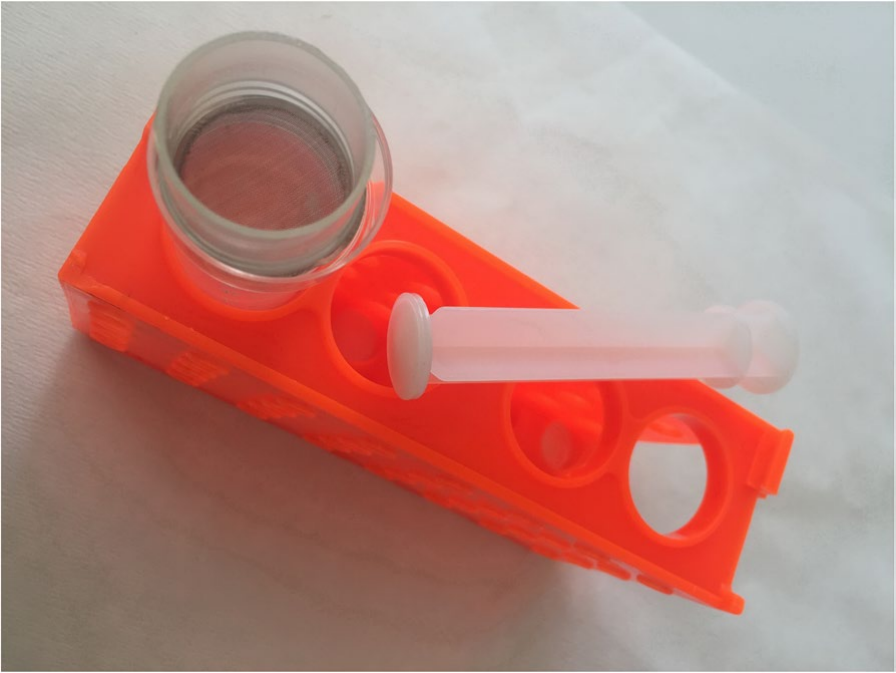
Image of the sieving device (100 μm cell strainer) for pulverization and filtration of ALN tissue to obtain cell suspension free from fat and connective tissue (for details see Methods)

Previous mechanical disaggregation procedures of ALN tissue involved chopping with scalpel blade and multiple injections [19], use of rotating knifes [15] and filtering through 100 μm cell strainer [16]. In our study, frozen ALN tissue specimens were processed for fractionation on ice in the following manner:

**a**. The ALN tissue samples were quickly thawed, the weight measured and then chopped with scalpel in a small glass petri dish; **b.** The pieces of ALN tissue were resuspended with PBS (4 × 1 mL) and simultaneously transferred on an INOX 18/10 sieve (mesh size 100 × 100 μm, net diameter ⌀ 20 mm) obtained from Fasil A.D. Arilje, Serbia, (www.fasil.co.rs) which is placed above a new petri dish (Fig. 6); **c.** Macerated tissue was pulverized on the sieve surface with rubber piston to separate fat and connective tissue from intact cells which are filtered through a mesh into a petri dish; **d**. The cell suspension was then transferred into an Ependorf tube, centrifuged (850 g, 15 min, 4 °C), and the cell pellet packed volume (V_pack_) estimated; **e.** The supernatant was decanted and the obtained cell pellet diluted with 5 x V_pack_ of ice-cold hypotonic lysis buffer and processed as described below (see Section *Protocol for fractionation of HeLa cells...*); **f**. An aliquot (50 μL) of pooled cell pellet was taken for viable cell counting with heamocytometer (Neubauer chamber) by Trypan blue exclusion. Further steps of fractionation of mALN cell suspension were identical as performed for HeLa cells (see below under: Steps 1-11)

### Hela Cells

HeLa cells were grown in RPMI 1640 medium supplemented with 10% Fetal Calf Serum (serum) in the CO2 incubator. For the time course experiment, 6 medium T flasks (dimensions: 75 cm^2^) were grown until 80-90% confluency. Then cell monolayers, in all flasks, were washed with PBS followed by addition of serum free RPMI which was left to incubate with the cells for 12 hour. At the time point “zero”, a moment after a 12-hour incubation ended, serum free RPMI was replaced by the working medium (RPMI plus 10% FCS) to initiate serum-treatment. Cell suspension from the “zero” flask was fractionated immediately and successive fractionations followed at the incubation time points of 2, 4, 5, 6 and 7 hours.

### Protocol for Fractionation of HeLa Cells and/or mALN Cell Suspension

Fractionation into nuclear and cytosolic extracts of HeLa cells and/or mALN Cell Suspension was prepared by the modifications of the protocols of Prusty et al. [9] and Riol et al. [32]. Considering the facts that the quoted references were used on primary tumor tissue and lymphocytes, respectively, we here describe the full fractionation protocol, adopted from Chemicon Inc. [21], which was used in this study:

1. After trypinization of the HeLa cell monolayer and determination of the cell pellet packed volume (V_pack_), the cell pellet was resuspended in 5xV_pack_ of ice-cold hypotonic lyses buffer (see below under *Extraction Solutions*); 2. For the purpose of cell swelling, the lysate was incubated on ice for 15 min, centrifuged at 850 g for 15 min at 4 °C, and supernatant discarded; 3 The cell pellet, from multiple ependorf tubes was again resuspended in 5xV_pack,_ pooled in one tube and recentrifuged at 850 g for 15 min at 4 °C, and supernatant discarded; 4. Subsequent cell pellet was resuspended in 2xV_pack_ of ice-cold hypotonic lyses buffer, and homogenized by drawing and ejecting the cell suspension content with a syringe/No 27 gauge needle; 5. Homogenate was centrifuged at 8.000 g for 20 min at 4 °C, and supernatant saved (containing cytosolic extract) and an aliquot (50 μL) was used to measure total cytosolic protein; 6. Remaining cytosolic extract was immediately aliquoted, snap frozen with liquid nitrogen and stored at − 70 °C for TGFβ1 ELISA analysis, within the shelf time of 4 months; 7. Remaining pellet (which contains the nuclear portion of the cell lysate) was resuspended on ice in 1x V_pack_ (the original cell pellet volume) in the nuclear extraction buffer (see below, under *Extraction Solutions*); 8. Nuclei were lysed (disrupted) by drawing and ejecting the content with a syringe/No 27 gauge needle, with addition of 1% Igepal CA-630 when necessary; 9. Homogenate was gently agitated for 30 min on ice and centrifuged at 18.000 g for 10 min at 4 °C; 10. Supernatant (which contains nuclear extract) was saved and an aliquot taken for total nuclear protein determination; 11 Remaining nuclear extract was immeditelly aliquoted, snap frozen with liquid nitrogen and stored at –70 °C for cFOS determination.

### Solutions and Reagents

Fractionation of both HeLa cells and ALN cell suspensions included the use of following buffers: A. Hypotonic cell lysis buffer: 10 mM HEPES pH 7.9, 1.5 mM MgCl_2,_ 10 mM KCl, 2.5 mM DTT, 0.1% Triton X-100, plus PKIC (Protein Kinase Inhibitor Cocktail); and B. Nuclear extraction buffer: 20 mM HEPES pH 7.9, 1.5 mM MgCl_2,_ 420 mMNaCl, 0.2 mM EDTA, 2.5 mM DTT, 1% Igepal CA-630, 25% (v/v) glycerol plus PKIC. In order to reduce proteolysis, dephosphorylation and denaturation of proteins, related inhibitors were added into the lysing buffers prepared as stock solutions of inhibitor cocktails: I. 50 x PIC (Protease Inhibitor Cocktail) in ethanol as solvent containing: 0.5 mg/mL leupeptin; 0.5 mg/mL pepstatin; 0.8 mg/mL benzamidine hydrochloride hydrate; and 0.1 M PMSF. The stock was aliquoted in 50 μL aliquots and kept at −20^o^ C. II. 100 x KIC (Kinase Inhibitor Cocktail) in water as solvent, contained: 0.5 M sodium fluoride (NaF); and 0.1 M sodium orthovanadate. The stock was aliquoted in 60 μL aliquots and kept at -20 °C. III. 40 x DTT (0.1 M DTT solution is used to reduce disulphide bridges in proteins. The stock was aliquoted in 100 μL aliquots and kept at -20 °C.

### Determination of Protein Yields and cFOS / TGF-β1 Protein Levels

Protein determination of nuclear and cytosolic protein yields (presented in Table 2) was performed in microplates by a micro Lowry assay [33] with the absorbance read at 650 nm (Bio Tek Instruments, Inc., Winooski, Vermont, USA). Final concentration of total proteins for both cytoplasmic and nuclear ALN tissue extracts covered the range from 3.2-9.9 mg/mL. However, the optimal total protein concentration of 2 mg/mL for TGF-β1 and 3 mg/mL for cFOS was used for comparative analysis within the single plate/assay (Fig. 5). The level of cFOS protein was determined by the Transcription Factor Assay Colorimetric kit according to the manufacturer’s instructions [21]. The results are presented as percentage of the absorbance at 450 nm compared to simultaneously analyzed cFOS positive control (4 hr. time point of serum treated HeLa nuclear extracts) considered as 100%. The TGFβ1 protein concentration was determined as described before [7]. The immunoreactive TGF-β1, obtained by acid-activation of latent TGF-β1, was analyzed by the Quantikine TGF-β1 ELISA kit according to the manufacturer’s protocol for the cell culture/Urine with RD1-21 diluting agent/ dilution factor: 14-24. (R&D Systems Inc. Minneapolis, MN, USA). Taken together, ELISA spectrophotometric analysis for the measurements of cFOS and TGF-β1, with the absorbances at 450 nm and 650 nm, was performed on the Microplate ELISA reader Wallac 1420 (PerkinElmer, Inc., Waltham, Massachusetts, USA). The cFOS / TGF-β1 levels in ALN extracts are presented in Table 2 and Fig. 5.

### Statistics

For statistical analysis, the Stat Soft (Hamburg, Germany) statistical package was used. The median values and one-tailed test were used, for ALN tissue extracts measurements, since the number of ALN samples was small [34]. The significance levels between the medians for the subgroup distributions of cFOS and TGFβ1 points in control versus metastatic nuclear and cytosolic ALN tissue extracts, respectively was calculated via the Chi-sqare, one-tailed test. Level of significance for statistical tests was set at *p* < 0.05. All experimental points in Figs. 2, 3, 5B and C were performed in duplicates. The variation between the duplicates in a single assay did not exceed 20%. The coefficients of variation (CV) for the cFOS and TGF β1 groups ranged from 22.74 to 70.21%.

## Data Availability

Not applicable.

## Abbreviations

BC: Breast Cancer
ALN: Axillary Lymph Nodes
mALN: Metastatic Axillary Lymph Nodes
nALN: Normal Axillary Lymph Nodes
FNCS: Fractionated Nodal Cell Suspension
TGFβ1: Transforming Growth Factor β1
H&E staining: Haematoxylin and Eosin staining
IHC: Immunohistochemistry
TNBC: Triple Negative Breast Cancer
FCS/serum: Fetal Calf Serum
PKIC: Protein Kinase Inhibitor Cocktail
